# An evaluation of menstrual health apps’ functionality, inclusiveness, and health education information

**DOI:** 10.1186/s12905-025-03812-1

**Published:** 2025-05-28

**Authors:** Emma Bucher, Callie Sharkey, Abby Henderson, Briette Basaran, Sarah Meyer, Chen X. Chen

**Affiliations:** 1 Indiana University School of Nursing, Indianapolis, IN USA; 2 University of Arizona College of Nursing, Office 439, 1305 N Martin Ave, Tucson, AZ 85721 USA

**Keywords:** Menstrual cycle, Menstrual symptoms, Menstrual period, Menstrual tracking, Dysmenorrhea, Mobile applications

## Abstract

**Background:**

Menstrual health apps have become increasingly popular, providing users with a tool to monitor and learn about menstrual cycles, symptoms, and management. While previous research examined different aspects of menstrual health apps (e.g., fertility tracking), few examined menstrual health apps comprehensively to examine the menstrual health apps’ functionality, inclusiveness, and health education information. The purpose of this study was to evaluate menstrual health apps’ functionality, inclusiveness, and health education information.

**Methods:**

In this descriptive study, two reviewers independently searched, screened, and evaluated each app using a standardized tool. Three terms (i.e., “period pain,” “period app,” and “menstrual cramp”) were used to search the Apple App Store. Apps were also cross-searched on the Google Play Store. We screened 60 apps. After excluding duplicates and apps that did not meet the inclusion criteria, 14 apps were evaluated on their functionality (user experience, internet and language accessibility, privacy, cycle-prediction, and symptom-tracking ability), inclusiveness (cycle lengths and regularities, fertility goals, and gender expressions and sexualities), and menstrual health education information (credibility and comprehensiveness, presence of additional health information, and information on when to seek care). We used a modified version of the Mobile App Rating Scale to score each app.

**Results:**

For functionality, half of the apps had third-party advertisements. Most (71.4%) did not require cellular connection to utilize menstrual symptom-tracking, and 71.4% shared user data with third parties. All had cycle-prediction and symptom-tracking functions. The mean number of relevant symptoms tracked was 17.5 (SD = 5.44). None of the apps used or cited validated symptom measurement tools. For inclusiveness, all apps could be tailored to cycle lengths other than 28 days, 85.7% had ovulation prediction functions, 50% had neutral or no pronouns, and 92.9% allowed users to input at least one contraceptive type. For health education information, 42.9% cited medical literature.

**Conclusion:**

This study suggests a lack of professional involvement and gender inclusivity in menstrual health app development. Healthcare professionals should educate themselves on apps’ functionality, inclusiveness, and health education information before recommending apps. Additional research is needed to understand diverse users’ perspectives on menstrual health apps.

**Supplementary Information:**

The online version contains supplementary material available at 10.1186/s12905-025-03812-1.

## Introduction

Menstrual health apps have gained immense popularity among users [1, 2]. These apps have become convenient tools for people who menstruate, enabling them to track their menstrual cycles and learn about menstrual health [1]. It is not surprising that these apps are widely used, considering that approximately 26% of the world’s population consists of people who menstruate [3].

Given the popularity of menstrual health apps among users, researchers have sought to examine their functionality**.** For example, multiple teams have evaluated the fertility-tracking features of menstrual health apps [4–9]. Some of these reviews solely focused on apps in the Apple App Store, without cross-referencing findings with the Google Play Store to ensure apps were also available to Android users [4]. Additionally, researchers have explored the potential of using menstrual cycle-tracking apps for epidemiologic research [10]. However, several gaps remain in the literature on menstrual health apps.

First, even though cycle-tracking and symptom-tracking are both important features for users [1, 11], studies have primarily focused on cycle-tracking rather than symptom-tracking functions. Symptoms like menstrual pain are often dismissed [12], thereby reinforcing the common belief that symptoms are part of a menstrual cycle that people should accept [13, 14]. However, menstrual health apps’ ability to monitor and document patterns in symptoms and cycle variations may aid in identifying potential menstrual health problems and help users recognize when to seek medical care for abnormal symptoms.

Second, there is scant research on privacy-related features of menstrual apps. Menstrual apps often require users to input personal and sensitive information, such as menstrual cycle dates, symptoms, and fertility and pregnancy-related data [15]. Users risk losing control over who has access to their data [15, 16]. Robust privacy features are critical to ensure that data remains confidential and protected from unauthorized access, sharing, and misuse. The importance of privacy has become increasingly evident due to several instances where personal data were sold or transferred without user consent or awareness [16]. Additionally, menstrual tracking data is hard to erase, and stored menstrual data may translate to targeted ads about fertility and menstruation appearing on personal devices [17]. Unfortunately, menstrual health data sharing is not well-regulated in many countries [16]. For example, in the United States, notice and consent forms often indicate that data sharing is legal, meaning that the onus of data privacy is on the app user rather than the app itself [16].

Third, few studies have examined menstrual health apps’ inclusiveness. Menstruating individuals are diverse in their cycle length, fertility goals, gender expression, and sexual orientation [18]. Thus, it is important to determine whether menstrual health apps accommodate diversity in cycle length, fertility goals, gender expression, and sexual orientation. Given the increasing number of menstrual health apps on the market, it may be difficult for users to identify apps that accurately and effectively address their personal needs and goals.

Fourth, there is limited research on the credibility of menstrual health information on apps. Menstrual health apps have the potential to provide menstrual health education to a significant number of people, some of whom may not have reliable access to formal healthcare. There are roughly 30 million uninsured Americans, making up approximately 8.3% of the population [19] who lack access to primary care, let alone gynecological care. While these apps do not replace gynecological care, they are easily accessible and may serve as a starting point for users who cannot or choose not to access formal healthcare for menstrual health problems. Evaluating the credibility of menstrual health information is important because while credible health information can empower users to make informed decisions and manage their menstrual health, misinformation can lead to inappropriate treatments or delayed medical intervention.

We sought to fill these gaps in the literature by evaluating menstrual health apps’ functionality, inclusiveness, and menstrual health information. While other studies focused on apps geared towards menopause or fertility tracking [4–7], we focused on several different aspects to provide a more comprehensive review of each app. Therefore, the purpose of this descriptive study was to evaluate menstrual health apps in terms of their functionality (including user experience, internet and language accessibility, privacy, and symptom-tracking), inclusiveness (including cycle lengths and regularities, fertility goals, and gender expressions and sexualities), and health education information (credibility and comprehensiveness, presence of additional health information, and information on when to seek care). The findings from this study will help inform app users, healthcare providers, and app developers. The findings from this study will help inform app users, healthcare providers, and app developers. These findings will assist app users in making informed choices, guide developers in creating user-centered and inclusive apps, and help healthcare providers recommend suitable apps to their patients based on individual needs.

## Methods

In this descriptive study, we searched, screened, and evaluated the functionality, inclusiveness, and health education information of menstrual health apps. Five of the six authors were Bachelor of Science in Nursing students who reviewed credible literature on mobile app reviews, menstruation, and menstrual symptom management in preparation for conducting this project. The senior author was a PhD and MBBS-prepared researcher with over 12 years of experience studying menstrual health. The senior author trained and advised the other authors in conducting this project and helped resolve questions or inconsistent ratings between student reviewers.

**Fig. 1:**
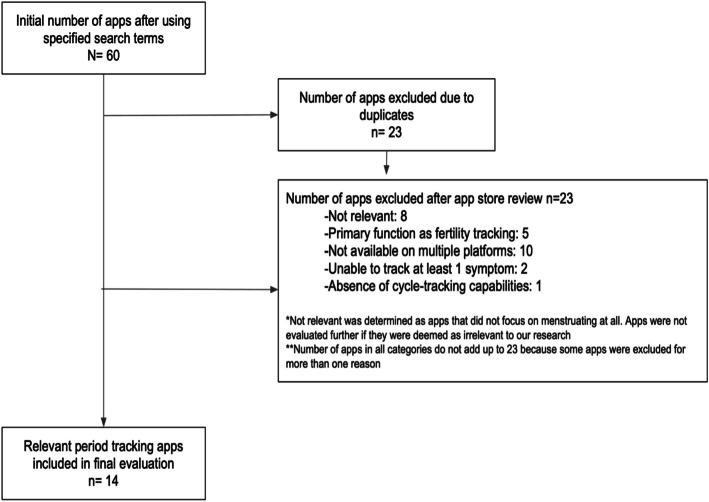
Flowchart for searching and screening of apps

Figure 1 is the flow chart of app searches and screening.

### Search strategy

Using an iPhone, authors searched the Apple App Store for menstrual health apps between September 28 th and November 16 th of 2022. The following search terms were used: period pain, period app, and menstrual cramp. We chose these search terms because they are popular terms used by the general public to search for information on menstrual pain and symptoms [20, 21]. Two authors independently searched the Apple App Store using one search term at a time, and any discrepancies in search results were resolved within the group. The first 20 results from each search were listed, resulting in 60 apps to screen for inclusion/exclusion. We listed the first 20 results from each search for two reasons. First, limiting the search allowed the authors to balance the feasibility and comprehensiveness of the review. Second, app store optimization statistics have shown that users only spend an average of 10.2 s on each app store search page before choosing an app to download [22], suggesting that most users download apps ranked highly.

### Screening inclusions/exclusion criteria

The list of 60 apps yielded some duplicate results (*n* = 23). After removing the duplicates, 36 unique apps were screened against the inclusion and exclusion criteria independently by two authors. Apps were included in the review if they were relevant to menstruation, free to download, available in English, able to track at least one menstrual symptom, able to track menstrual cycles, and available on both the Apple Store and Google Play. We cross-searched 36 unique apps on the Google Play Store and included apps available in both the Apple and Google app stores to ensure apps were accessible to users with different devices. Apps were excluded if they met one or more of the following exclusion criteria: the app’s primary function was menopause-tracking or fertility-tracking, the app was designed for men and/or non-menstruating partners, and/or the app was designed for healthcare providers only. Any discrepancies were discussed and resolved between the two authors. When needed, a third author helped reach a final consensus. As shown in Fig. 1, 14 apps met the inclusion criteria.

### Data extraction tool and process

Our team developed a standardized data extraction tool based on our evaluation criteria. All authors pilot-tested and revised the extraction tool. For each app, we extracted data on functionality, inclusiveness, and health education information. Two authors independently extracted data from each app between October 2022 and February 2023. Any discrepancies were resolved with discussion within the group.

#### Functionality

Four aspects of functionality were evaluated, including (1) user experience, (2) internet and language accessibility, (3) privacy, and (4) symptom-tracking ability. To assess user experience (i.e., overall experience a person has when interacting with the app), we examined the presence of promoted or required in-app purchases, third-party advertisements, the overall ease of using the app, the appropriateness of the app’s arrangement (i.e., appropriateness of the size of buttons, icons, and sections), the app’s visual appeal, and the customizability of settings. For accessibility, we evaluated internet requirements and language accessibility. Specifically, we downloaded and opened each app in airplane mode to determine if the internet was required when using the cycle and symptom-tracking functions, and we looked in the app store to see if the apps were available in languages other than English. To assess each app’s privacy features, we reviewed their privacy policies, noting password protection options, the ability to customize privacy settings, the ease of accessing the privacy policy, and details about data sharing. Lastly, symptom-tracking abilities were assessed by counting and recording the number of relevant symptoms listed in each app and the symptom rating scale used (e.g., presence/absence, severity). We noted whether the app provided a visual display of symptom patterns over time. Additionally, we reviewed the app descriptions and references cited in each app and examined specific symptom measurements against the menstrual symptom literature to determine whether they had been validated by research.

#### Inclusiveness

We assessed inclusiveness by evaluating each app’s ability to account for diverse (1) cycle lengths and regularities, (2) fertility goals, and (3) gender expressions and sexualities. The inclusiveness of cycle lengths and regularities was determined by the app’s ability to enter and tailor cycle lengths other than the typical 28-day cycle. We looked at whether the app had the function present for a user to input their most recent cycle dates. This was evaluated by entering three previous cycle dates based on a typical 28-day cycle. We also noted if apps allowed users to manually enter cycle lengths, as opposed to the app predicting the length for the user. To assess inclusiveness of fertility goals, we examined whether apps offered ovulation prediction and an option to input contraception methods. If an app allowed users to input contraceptive options, the number and types of contraceptive methods were extracted. To assess inclusiveness of gender expression and sexuality, we evaluated the overall neutrality/customizability of the pronouns used in the app and the availability of LGBTQ + content.

#### Health education information

Three areas of health education information were evaluated including, (1) credibility and comprehensiveness of menstrual health education, (2) presence of additional information on hormonal changes throughout the cycle, and (3) information on when to seek care. First, we looked at the reliability of the menstrual health information, the comprehensiveness of the information, if the information came from a credible source, and at the clarity and accuracy of the charts, graphs, images, and/or videos. Specifically, for comprehensiveness, we assessed whether the menstrual health information covered a wide range of topics, including menstrual cycle phases, symptoms, management strategies, and potential health issues. For credibility, we evaluated whether the information was sourced from reputable organizations or publications and looked for citations and references within the app content to ensure it was evidence-based. We assessed the clarity and accuracy of charts, graphs, images, and videos by checking if visuals accurately reflected the underlying information without distortion or misrepresentation, and whether the data sources were credible and properly cited. We used a scoring system as described in Table 1. Second, we assessed if apps had a presence of additional health information on hormonal changes throughout the menstrual cycle. Lastly, we determined whether the apps provided educational information on when to seek professional care for menstrual symptoms and whether the apps provided a recommended treatment for menstrual symptoms.

**Table 1:** App evaluation criteria and scoring

Broad Criteria	Specific Criteria	Items	Scale/Scoring
Functionality	User Experience	1. Third-party ads 2. Ease of learning to use/navigate app 3. Appropriate arrangement/size of buttons/icons 4. Visual appeal 5. Ability to customize the settings and preferences	1. 0 = ads present, 1 = no ads 2. 0–4 (the higher, the easier to navigate) 3. 0–4 (the higher, the more appropriate) 4. 0–4 (the higher, the more visually appealing) 5. 0–4 (the higher, the more customizable)
	Internet and Language Accessibility	1. Need for cellular connection 2. Availability of other language versions	1. 0 = connection required, 1 = no connection required 2. 0 = no additional languages, 1 = one additional language, 2 = more than one additional language
	Privacy	1. Password protection option 2. Location of privacy policy	1. 0 = no, 1 = yes 2. 0 = no policy located, 1 = hard to locate policy, 2 = easy to locate policy
	Symptom Tracking	Relevant symptoms tracked	0 = 0 symptoms 1 = 1–5 symptoms 2 = 6–10 symptoms 3 = 11–15 symptoms 4 = 16–20 symptoms 5 = 21 + symptoms
Inclusiveness	Cycle Length	Ability to tailor to cycle length other than 28 days	0 = not able to tailor to cycle length other than 28 days 1 = able to tailor to cycle length other than 28 days
	Fertility goals	1. Ovulation date prediction 2. Option to input contraception methods	1. 0 = does not predict ovulation date 1 = predicts ovulation date 2. 0 = 0 methods 1 = 1–2 methods 2 = 3–4 methods 3 = 5–6 methods 4 = 7–8 methods 5 = 9 + methods
	Gender expression and sexuality	1. Inclusivity of the app’s pronouns used 2. Availability of LGBTQ + content	1. 0 = Non inclusive pronouns 1 = Inclusive pronouns 2. 0 = no LGBTQ + content 1 = additional LGBTQ + content
Health Education Information	Credibility and comprehensiveness of menstrual health education	1. Reliability of menstrual health education information 2. Comprehensiveness of information 3. Information from a credible source 4. Clarity and accuracy of charts/graphs/images/videos	1. 0–5 (the higher, the more reliable) 2. 0–5 (the higher, the more comprehensive) 3. 0–5 (the higher, the more credible) 4. 0–5 (the higher, the more accurate/clear)
	Presence of additional health information	Additional information about hormonal changes throughout the menstrual cycle	0 = no additional information 1 = information on hormonal changes
	Information on when to seek care	Discussion on when to seek professional care for menstrual symptoms	0 = does not discuss when to seek professional care 1 = does discuss when to seek professional care

### Scoring and data analysis

Two authors independently scored each app’s functionality, inclusiveness, and health education information using our adapted version of the Mobile Application Rating Scale [23], a generic tool designed to evaluate mobile apps. Table 1 shows the evaluation criteria upon which each app was scored. From the original Mobile Application Rating Scale, we removed less relevant questions and added questions related to our evaluation criteria. Specifically, we added scoring questions about internet and language accessibility, privacy, menstrual cycle tracking, symptom tracking, inclusiveness, and relevant health education information. With regard to scoring symptom tracking, we decided to count symptoms that changed throughout the menstrual cycle rather than symptoms unrelated to menstruation. These symptoms were not grouped into specific categories (e.g., menstrual symptoms, premenstrual symptoms, menopausal symptoms) because certain symptoms (e.g., sleep problems) can fall under multiple categories. Table 2 shows the symptoms included. High overall scores on the adapted Mobile Application Rating Scale suggest an app is highly functional, aims to be inclusive, and provides quality menstrual health information. The highest possible score was 59 points. Descriptive statistics (mean, standard deviation, frequency, and percentage) were used to summarize the scores.

**Table 2:** Symptoms assessed in included apps

Menstrual Symptoms	Number of Apps Tracking the Symptom	Percentage of Apps with the Symptom
Menstrual Cramps	14	100.0%
Headache/Migraine	14	100.0%
Swollen/Tender Breasts	13	92.9%
Backache/Back Pain	12	85.7%
Bloating	12	85.7%
Change in Appetite/Cravings	12	85.7%
Sad/Depressed/Depressive Symptoms	12	85.7%
Sleep Problems	12	85.7%
Menstrual Heaviness	12	85.7%
Nausea	11	78.6%
Diarrhea	11	78.6%
Anxiety	11	78.6%
Fatigue	11	78.6%
Acne	11	78.6%
Constipation	10	71.4%
Irritability/Anger	10	71.4%
Sex Drive/Interest	10	71.4%
Cervical/Vaginal Discharge	9	64.3%
Pain All Over/Body Aches/Muscle Pain	6	42.9%
Poor Concentration	5	35.7%
Hot Flashes	5	35.7%
Swelling	4	28.6%
Chills	4	28.6%
Night Sweats	4	28.6%
Pelvic Pain (beyond menstruation)	3	21.4%
Painful Urination	2	14.3%
Vomiting	2	14.3%
Painful Sex	1	7.1%
Increased Bowel Frequency	1	7.1%
Decreased Bowel Frequency	1	7.1%

## Results

**Fig. 2:**
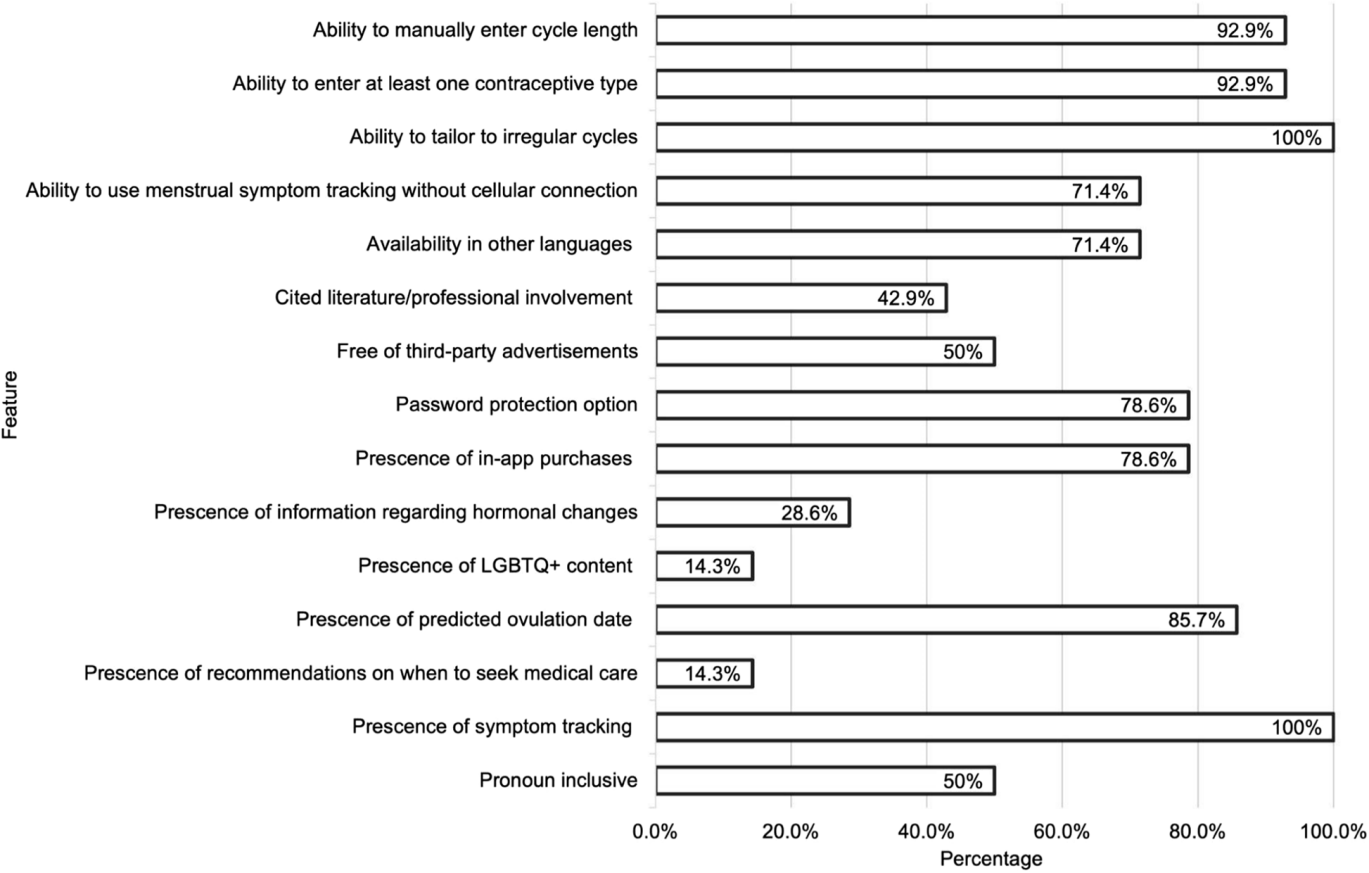
Percentage of apps with selected features (*N* = 14)

Figure 2 shows the percentage of apps with select functionality, inclusiveness, and health education information features. Below we describe the characteristics and ratings of the apps’ functionality, inclusiveness, and health education information.

### Functionality

#### User experience

Third-party advertisements were found in half of the apps. Three apps were entirely free and did not have the option to make in-app purchases. The other apps had in-app purchases ranging from $2.99 to $99.99 for annual subscriptions to premium versions of the apps. Premium versions of each app claimed to remove in-app advertisements and add other features like educational material or the option to personalize the app by changing the app’s color scheme.

On the adapted Mobile Application Rating Scale, ease of use had a mean score of 2.86 out of 4, appropriateness of the app’s arrangement (i.e., appropriateness of the size of buttons, icons, and sections) had a mean score of 3.36 out of 4, visual appeal had a mean score of 2.71 out of 4, and customizability had a mean score of 3.14 out of 4 possible points.

#### Internet and language accessibility

Three of the 14 apps (21.4%) required cellular data to use the cycle and symptom-tracking functions. Most apps (71.4%) had additional languages available besides English. The languages available ranged from only English to 30 other language options. Accessibility in other languages had a mean score of 1.29 out of 2 possible points.

#### Privacy

Most apps (78.6%) had an option for users to set up a password. The mean score for password protection was 0.79, with a score ranging from 0 to 1 point. Only one app (i.e., *Flo Anonymous Period Tracker)* had an “anonymous mode” option for users. The app claimed that under the anonymous mode, it would not sell users’ personal information (e.g., email, identifiers) to third parties and users could access, modify, and erase their data by contacting the UK-based company. This was the only app the authors evaluated that allowed users to customize their privacy settings.

All apps contained privacy policies. Seven had privacy policies in the app settings and on the company’s website. The mean score for ease of locating the app’s privacy policy was 1.79 out of 2. Privacy policies ranged from simple bolded statements such as “We do not and will not sell your data” from *Stardust Period Tracker* to more comprehensive policies that outlined data elements that could be shared and whether users could opt out. Regarding data sharing, 71.4% of the apps shared user data with third-party sources and advertisers. Eight apps (57.1%) shared unidentifiable information, such as the type of device and app usage data that does not identify the user, and five apps shared identifiable information (e.g., the user’s name and age) with third parties. Two apps indicated that they shared the user’s location with law enforcement if subpoenaed, and one app shared the user’s location with third parties. Half of the apps required users to agree to the privacy policy before using it, and five (35.7%) required users to make an account through their email.

#### Symptom-tracking ability

Table 2 lists the number and percentage of apps tracking menstrual symptoms. The mean number of relevant menstrual symptoms tracked by the reviewed apps was 17.5 (SD = 5.44). All apps allowed users to track menstrual cramps and headache symptoms. A large majority (85.7%) of the apps could track backache and bloating symptoms, while only one app could track increased bowel frequency, decreased bowel frequency, and painful sex symptoms. Most apps also included swollen or tender breasts, changes in appetite, sleep problems, menstrual heaviness, nausea, diarrhea, fatigue, acne, constipation, changes in sex drive, and cervical and vaginal discharge symptoms. Mood symptoms such as depressive symptoms, anxiety, and irritability were in more than half of the apps (See Table 2). On the adapted Mobile Application Rating Scale, symptom tracking had a mean score of 3.86 out of 4.

Apps used varied strategies to measure symptoms. For instance, some apps (*n* = 11) only allowed users to input the presence or absence of symptoms, while other apps (*n* = 3) allowed users to input one of three symptom severity levels. Half of the apps (*n* = 7) displayed a visual figure for users to track their symptoms over time. None of the apps had professional involvement or cited sources within their symptom trackers.

### Inclusiveness

#### Cycle lengths and regularities

A large majority of apps (92.9%) allowed users to enter their cycle length, and all apps (*n* = 14) included cycle-tracking that could be tailored to users with cycle length other than 28 days.

#### Fertility goals

All but two apps (85.7%) had an ovulation prediction function. However, some apps (14.2%) required the user to go into settings and turn on ovulation prediction and fertility planning functions, depending on the user’s personal fertility goals. Only one app did not have an option for entering contraception methods. While 92.9% of apps allowed users to enter at least one type of contraceptive, a large majority (78.5%) of the apps allowed an input of a contraception method besides an oral contraceptive pill. The number of contraception method options listed ranged from only one (i.e., contraceptive pill) to more than 15. The *Period Tracker-Eve* app had the most (*n* = 17) contraception methods from which users could choose. On the adapted Mobile Application Rating Scale, contraceptive methods had a mean score of 2.64 out of 5 points.

#### Gender expressions and sexualities

Half of the apps (50%) used gender inclusive pronouns (if a user was able to input preferred pronouns or the app did not use pronouns). One app (Ovia fertility, period tracker) had an option for users to identify as LGBTQ + during app setup. Only two apps included LGBTQ + content related to menstrual health. The *Period Tracker-Eve* app had education and community groups tailored to LGBTQ + menstrual health.

### Health education information

#### Credibility and comprehensiveness of information and visuals

Six of the reviewed apps (42.9%) cited literature on menstrual health. The mean score for access to reliable menstrual health education information was 2.21 out of 5 possible points. The mean score for comprehensiveness of information was 2.36 out of 5 possible points. For clarity and correctness of charts and graphs, the mean score was 2.21 out of 5 points. For credibility of sources within the apps, there was a mean score of 2.57 out of 5 points.

#### Presence of additional health information

Four apps (28.6%) included information on hormonal changes during the menstrual cycle.

#### Information on when to seek professional care

*Cycle and Period Tracker-Femia* and *Period Tracker Eve* were the only two apps that encouraged users to seek medical attention for their menstrual symptoms.

#### Information on menstrual symptom treatments

For menstrual symptom treatments, 5 apps (35.7%) included educational information on treatments on their free versions. Among these 5 apps, 4 had cited literature. However, only one app (*Always You: Period Tracker)* recommended evidence-based first line treatments for menstrual pain (i.e., NSAIDs). *Always You: Period Tracker* was also the only app to provide personalized suggestions based on the symptoms users selected. The most commonly recommended treatments were dietary adjustments (35.7%), lifestyle changes (e.g., physical activity, sleep) (21.4%), dietary supplements or natural products (21.4%), rest or relaxation (14.2%), mind–body approach (e.g., yoga) (14.2%), and over-the-counter medication (14.2%).

### Total scores of included apps

**Fig. 3:**
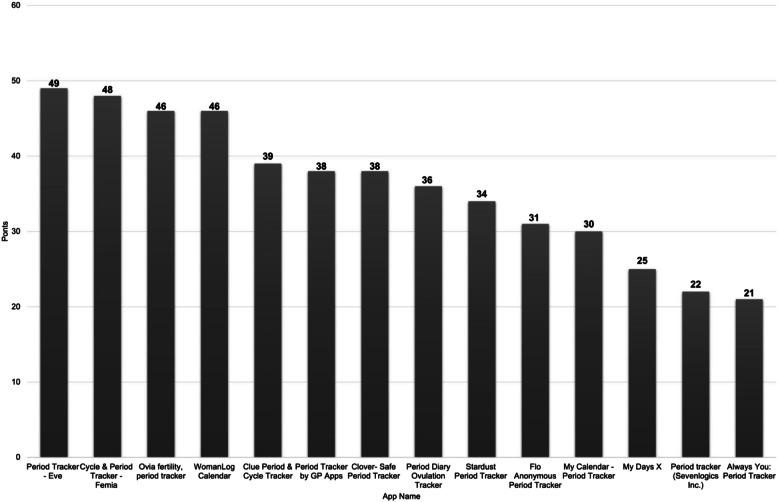
Ranking of apps by total points (possible points: 0–59)

Figure 3 shows the total score for each app. The mean score for all reviewed apps was 35.93, with a standard deviation of 9.35. *Period Tracker-Eve* scored the highest with 49 points. Supplementary Table 1 shows the breakdown of scores for each app based on scoring criteria.

## Discussion

Mobile apps can be a convenient tool for menstruating individuals to track menstrual health and access relevant health education information [1]. With so many apps on the market, it is important to evaluate them to inform app users, healthcare providers, and app developers. We evaluated menstrual health apps using a standardized extraction tool and scored each app using a Mobile Application Rating Scale adapted based on our evaluation criteria. While other research studies have reviewed menstrual health apps [4–8, 10, 18], we adopted more comprehensive review criteria to capture various aspects of functionality, inclusiveness, and health education information.

Unlike previous studies of menstrual health app functionality that only reviewed fertility tracking or menstrual symptom tracking, we examined functionality more broadly by evaluating apps’ user experience, internet and language accessibility, privacy, cycle-tracking ability, and symptom-tracking ability. Consistent with a recently published paper [24], we found that most apps only captured the presence or absence of symptoms without collecting information on symptom severity. It is worth noting that no evidence of health professional involvement in the development of their symptom trackers was found. In addition, despite the availability of scientifically validated pain and symptom tools, none of the apps used these symptom measurement tools within their symptom trackers [24]. For privacy features, we found that data sharing was very common among apps. However, information about data-sharing policies was not always easily accessible and understandable. A previous study suggested that the readability of popular menstrual health apps’ privacy policies was problematic [25]. According to our study, some apps share identifiable information (e.g., name, location, emails) and/or other sensitive data. Given that some data-sharing policies are not easily comprehensible, it is unclear if users are fully aware of what is being shared.

This study provides insight into menstrual health apps’ inclusiveness. We found that some apps were more gender-inclusive, while other apps assumed all users self-identified as women. For example, some apps only used “she/her” pronouns. In another study reviewing 17 menstrual apps, researchers found that over 80% of the apps assumed users were cis-gendered [26]. In a qualitative study with menstrual health app users, participants voiced concerns about gender representation in menstrual health apps [27]. We found that some apps were more inclusive than others in regard to contraceptive methods and fertility goals. Given that menstrual health app users are diverse in reproductive goals and gender expression, apps need to be gender-sensitive and responsive to users’ varying needs and preferences.

Our findings expand upon current literature, highlighting concerns about the credibility of health education information in apps. We found that less than half of the apps included information with cited literature. In two other studies involving a larger number of apps, authors found an even smaller percentage of apps (1.8% and 14%, respectively) with a clear reference to health professionals or health researchers [8, 24]. Similar to our study, one study found that very few apps involved healthcare providers and experts in the development of the apps [24]. The lack of healthcare researchers or professional involvement has also been reported in other mobile health apps for the general public [28]. Misinformation on the apps could lead to inappropriate treatments or delayed medical intervention.

This study had several strengths. First, we reviewed multiple aspects of each app, including functionality, inclusiveness, and health education information. Second, we used multiple search terms to capture the most relevant apps for menstrual cycle and menstrual symptom tracking. We also ensured the apps were available on both the Apple App Store and Google Play Store. Third, two authors searched, screened, and reviewed each app independently to reduce bias in our review of the apps and enhance the reliability of our study.

Our study also had limitations. First, we only searched for apps in English in the U.S. Apple App Store and Google Play Store. While we gave additional points to apps that could be used in more than one language (as part of the language accessibility feature of functionality), we evaluated only the English version of each app. Second, we only searched app stores using three search terms. Additional search terms may identify apps that we missed. Third, we only reviewed the free version of each app; however, this was consistent with our original intent to review apps that were accessible to the largest number of users. Fourth, although our search initially yielded 60 results, we only reviewed a small number of apps after excluding duplicate apps and those that were not relevant to our study. Focusing on a small number of apps allowed us to provide an in-depth review of each app, using our comprehensive evaluation criteria. Fifth, we did not include diverse app users as reviewers for the apps. This could have provided a more well-rounded review, as research on mental health apps shows disagreement between professional and consumer reviews [29], though it is unclear if the same applies to reviews of menstrual health apps. A professional’s opinion does not inherently qualify an application to be better than another, as every user has different lived experiences and preferences. Sixth, we did not evaluate how easily individuals with various abilities (e.g., visual, auditory, motor, or cognitive impairments) can use and interact with the app as part of the accessibility evaluation. Seventh, while we reviewed whether an app could be tailored to cycle lengths other than 28 days, we did not test apps for compatibility with various menstrual irregularities beyond longer or shorter cycle lengths. Other researchers found that menstrual apps cannot make sense of irregularity [18]. People with menstrual irregularities have reported finding apps challenging, and the inability to cater to irregularity has been cited as a reason for stopping use, feeling frustrated, or even experiencing self-blame [1, 30]. Finally, for inclusiveness, we did not review content and features related to age and cultural factors. Menstrual health needs and preferences can vary significantly across different age groups and cultural backgrounds. A previous study suggested that most menstrual apps are designed primarily for adults, with very few targeting new menstruators. Additionally, no apps had built-in support to assist individuals who are perimenopausal or menopausal. [26]. Attitudes about pain and menstruation, preferences for app functions, and needs for menstrual health information may vary based on age and culture.

The findings of this study have multiple implications for future research. Researchers need to rigorously test menstrual health apps among diverse users, assessing apps’ functionality, inclusiveness, and menstrual health education information. This could include assessing the cycle prediction function, user experience, and users’ understanding of privacy policies. In addition, future studies should analyze how apps cater to different ages and cultures. Conducting surveys and qualitative interviews to gather data on user experiences can provide insights into user needs and preferences, informing app design and feature development to make apps age-appropriate and culturally sensitive. Furthermore, menstruation for transgender and gender-diverse individuals can cause distress [31]. Thus, more research is needed on these communities’ perceptions and preferences related to menstrual health apps.

This study also has implications for clinical practice. Findings can inform healthcare providers about menstrual health apps to recommend to patients and factors to consider when providing recommendations (e.g., functionality, inclusiveness, and health education information). We generated a total score for each app (Fig. 3) and included a supplementary table showing the breakdown of scores for each app based on our evaluation criteria. Providers can recommend appropriate apps based on the needs of their patients.

Lastly, app developers need to keep their audience in mind and make the apps user-friendly. Additionally, users are more likely to trust the app if scientific sources are cited [32]. Getting input from researchers and healthcare providers specialized in menstrual health would enhance the credibility and trustworthiness of the apps. When developing and refining apps, developers should consider inclusivity in terms of users’ age, reproductive goals, contraceptive methods, sexual orientation, and gender expression. This can include allowing users to customize and personalize the app by choosing pronouns, backgrounds, and colors while also tailoring health education information to users’ unique needs.

## Supplementary Information

Supplementary Material 1

## Data Availability

The data that support the findings of this study are available from the corresponding author on reasonable request.
